# Surface-Initiated Photoinduced Iron-Catalyzed Atom Transfer Radical Polymerization with ppm Concentration of FeBr_3_ under Visible Light

**DOI:** 10.3390/ma13225139

**Published:** 2020-11-14

**Authors:** Monika Słowikowska, Kamila Chajec, Adam Michalski, Szczepan Zapotoczny, Karol Wolski

**Affiliations:** 1 Faculty of Chemistry, Jagiellonian University, Gronostajowa 2, 30-387 Kraków, Poland; slowikowska@chemia.uj.edu.pl (M.S.); kamila.chajec@spoko.pl (K.C.); michadam@cbmm.lodz.pl (A.M.); zapotocz@chemia.uj.edu.pl (S.Z.); 2 Center of Molecular and Macromolecular Studies, Polish Academy of Sciences, Sienkiewicza 112, 90-363 Lodz, Poland

**Keywords:** polymer brushes, photoinduced ATRP, iron-catalyzed ATRP, sacrificial initiator, grafting density, PMMA

## Abstract

Reversible deactivation radical polymerizations with reduced amount of organometallic catalyst are currently a field of interest of many applications. One of the very promising techniques is photoinduced atom transfer radical polymerization (photo-ATRP) that is mainly studied for copper catalysts in the solution. Recently, advantageous iron-catalyzed photo-ATRP (photo-Fe-ATRP) compatible with high demanding biological applications was presented. In response to that, we developed surface-initiated photo-Fe-ATRP (SI-photo-Fe-ATRP) that was used for facile synthesis of poly(methyl methacrylate) brushes with the presence of only 200 ppm of FeBr_3_/tetrabutylammonium bromide catalyst (FeBr_3_/TBABr) under visible light irradiation (wavelength: 450 nm). The kinetics of both SI-photo-Fe-ATRP and photo-Fe-ATRP in solution were compared and followed by ^1^H NMR, atomic force microscopy (AFM) and gel permeation chromatography (GPC). Brush grafting densities were determined using two methodologies. The influence of the sacrificial initiator on the kinetics of brush growth was studied. It was found that SI-photo-Fe-ATRP could be effectively controlled even without any sacrificial initiators thanks to in situ production of ATRP initiator in solution as a result of reaction between the monomer and Br radicals generated in photoreduction of FeBr_3_/TBABr. The optimized and simplified reaction setup allowed synthesis of very thick (up to 110 nm) PMMA brushes at room temperature, under visible light with only 200 ppm of iron-based catalyst. The same reaction conditions, but with the presence of sacrificial initiator, enabled formation of much thinner layers (18 nm).

## 1. Introduction

The reversibly deactivation radical polymerizations (RDRP) such as atom transfer radical polymerization (ATRP), nitroxide-mediated polymerization (NMP), reversible addition−fragmentation chain transfer (RAFT), and photoiniferter-mediated polymerization (PIMP) are the first choice methods for synthesis of various functional polymeric materials with advanced architectures such as molecular brushes [1,2], star-like polymers [3,4], polymer brushes [5,6,7,8], etc. RDRP methods enable controlled synthesis of polymers that are characterized by low dispersity, chain-end fidelity, and predefined molecular weight.

**Scheme 1:**
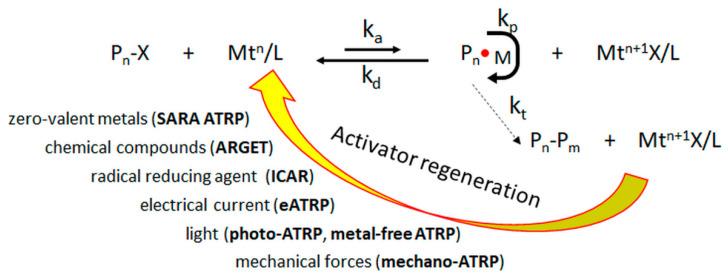
General scheme of externally controlled atom transfer radical polymerization (ATRP) methods.

The controlled character of ATRP is based on activation and deactivation of the growing radicals, which is realized by reversible halogen transfer from initiator to activating complex [9]. The classical ATRP requires the use of substantial amounts of catalyst to maintain the control over the reactive radicals. However, most of biological and electronic applications are highly restricted to the presence of impurities coming from transition metals. Therefore, significant scientific efforts have been devoted to development of new ATRP techniques, in which concentration of transition metal complex is greatly limited, due to regeneration of activator by the use of: radical chemical reducing agent in initiators for continuous activator regeneration (ICAR) ATRP [10], chemical reductants in activators regenerated by electron transfer (ARGET) ATRP [11], zero-valent metals in supplemental activator and reducing agent (SARA) ATRP [12], electrochemical reactions in electrochemically mediated ATRP (*e*ATRP) [13], light in both metal-free [14] and photoinduced ATRP (photo-ATRP) [15], or mechanical forces in mechanically induced ATRP (mechano-ATRP) [16] (see Scheme 1). Moreover, copper catalysts could be effectively replaced by iron compounds because of their lower toxicity and costs [17].

Surface-initiated ATRP techniques have been also successfully used for controlled decoration of various organic and inorganic surfaces leading to: homopolymer [18], copolymer [19], ladder-like [5], loop [20], gradient [21], or mixed brushes [22]. Surface-initiated ATRP techniques that use ppm amount of catalyst very often need addition of sacrificial initiator [23], which is required to maintain the control over the process, as low concentration of initiating groups on planar surfaces is not sufficient for effective regeneration of deactivator and shifting K_ATRP_ (k_a_/k_d_) towards deactivation process. Flejszar et al. have shown that surface-initiated eATRP with ppm amounts of copper catalyst (6–25 ppm Cu) in the presence of sacrificial initiator could be used for controlled synthesis of poly(2-hydroxyethyl acrylate) brushes [24]. However, the same polymerization conditions, but without free initiator in the solution were inefficient and enabled formation of only very thin brushes. In case of classical surface-initiated ATRP, controlled brush synthesis (without additional initiator) could be achieved by the addition of 5–25 mol% of deactivator (Mt^n+1^X/L) with respect to the activator (Mt^n^/L) [25,26].

An increasing attention is associated with the utilization of light-mediated polymerizations for syntheses of polymer brushes [27,28,29,30]. Both metal-free and photo-ATRP were found to be very efficient as they easily enable formation of patterned brushes [28,31,32] in an air-tolerant condition. Photo-ATRP techniques mostly use copper complexes often incompatible with biological applications or expensive iridium-based catalysts that are unlikely to be applied in the large-scale production as iridium is one of the rarest elements in the Earth. Furthermore, Yan et al. have recently shown that copper-based photo-ATRP could be effectively controlled only at high catalyst concentration (1100 ppm with respect to the monomer) [30]. It is expected that iron compounds due to their low toxicity and cost could be a good alternative in terms of biological applications. The problems with copper toxicity and its high concentrations are not significant for polymer brushes grafted from planar surfaces, as such materials could be easily purified. However, the developed conditions for planar surfaces could be easily transferred to some more challenging porous substrates or nano/microparticles where such issues are crucial. Moreover, there are many reports showing that iron catalysts could initiate and effectively mediate the photo-Fe-ATRP without any additional initiators [33] or even ligands [34], allowing application of simplified reaction setups. Utilization of iron-mediated ATRP in synthesis of polymer brushes was rather scarcely reported. There are only a few reports, including surface-initiated AGET [35] and Fe-mediated [36] polymerizations. However, to the best of our knowledge, there are no reports concerning surface-initiated photoinduced iron-catalyzed ATRP (SI-photo-Fe-ATRP). 

We report here SI-photo-Fe-ATRP with ppm amount of catalyst (200 ppm) for controlled synthesis of poly(methyl methacrylate) (PMMA) brushes under visible light. The current research was inspired by the paper of the Matyjaszewski group [37], who for the first time presented photo-Fe-ATRP in solution with ppm concentration of the catalyst. We have compared here the kinetics of SI-photo-Fe-ATRP with photo-Fe-ATRP. Furthermore, an effect of the addition of the sacrificial initiator on the brush growth was investigated. The grafting densities were calculated by analyzing the swelling ratio of PMMA brushes in toluene and determination of average molecular weights of free polymers generated in the solution. 

## 2. Materials and Methods 

### 2.1. Materials

Silicon wafers were obtained from ON Semiconductor (Roznov, Czech Republic). Hydrogen peroxide (30%, p.a.), sulfuric acid (96%, p.a.), hydrochloric acid (35–38%, p.a.), toluene (p.a.), tetrahydrofuran (THF, p.a.), dichloromethane (DCM, p.a.), ethanol (p.a.), and methanol (p.a.) were purchased from Chempur (Piekary Slaskie, Poland). In addition to this, (3-aminopropyl) trimethoxysilane (APTES, 99%), α-bromophenylacetyl chloride (BPA-Cl, technical, 80%), triethylamine (TEA, puriss. p.a., ≥99.5%, GC), ethyl α-bromophenylacetate (EBPA, 97%), and methyl methacrylate (MMA, 99%, ≤30 ppm MEHQ as an inhibitor) were purchased from Sigma Aldrich. Anisole (>99%), tetrabutylammonium bromide (TBABr, 99%) were purchased from Fluorochem (Hadfield, UK). Iron (III) bromide (FeBr_3_, anhydrous, >98%) was obtained from Alfa Aesar and stored in an argon atmosphere. MMA was passed through the basic alumina column to remove inhibitors before the polymerization, while anisole was passed through a neutral alumina column and dried over molecular sieves under argon atmosphere. Rest of chemicals were used as received. 

### 2.2. Methods

LED lamp emitting at 450 nm (M450LP1, Output Power 1850–2100 mW, Thorlabs, Newton, MA, USA) equipped with an aspheric condenser lens (Ø 50.8 mm, f = 32 mm, NA = 0.76, uncoated) and a large heat sink was used for photo-Fe-ATRP and SI-photo-Fe-ATRP. The specially designed reaction vessel was in contact with an aspheric condenser lens (it provides spatially homogeneous illumination) in order to maximize the light intensity and keep a constant distance from the LED light source in each polymerization (see Figure S1). The UV–VIS spectra were collected on a Varian Cary 50 UV-VIS spectrophotometer (Santa Clara, CA, USA) in transmittance mode. Atomic force microscopy (AFM) images were obtained using Dimension Icon AFM (Bruker, Santa Barbara, CA, USA). AFM was operating in the PeakForce QNM^®^ mode. ScanAsyst-air probes with a nominal spring constant of 0.4 N/m were used for topography measurements in air, while HQ:NSC36/Cr-Au probe (tip radius 30 nm, Mikromasch, Sofia, Bulgaria) was used for measurements in toluene. The spring constant of the HQ:NSC36/Cr-Au probe (0.97 ± 0.05 N/m) used for nanoindentation measurements was calibrated by the thermal-noise method. The deflection sensitivity of the optical beam-detection system was calibrated on nondeformable sapphire glass surface. The Young’s moduli were determined by fitting a Hertzian model to the approach curves using NanoScope Analysis software (Bruker, Santa Barbara, CA, USA). The values of Young’s moduli are shown as mean values ± standard deviation. In order to measure the brush thickness, the sample was scratched gently by tweezers, rinsed with copious amount of THF, ultrasonicated in toluene (5 min), and then, the AFM images were captured in an ambient air or toluene at the edge of the scratch. The average dry or wet brush thicknesses were determined by measuring the step height at several locations at the sample. FTIR spectra of the APTES-PBA monolayer grafted on the ITO surface were obtained by a Nicolet iS10 FT-IR spectrometer (Thermo Scientific™, Waltham, MA, USA) equipped with a grazing-angle reflectance accessory using p-polarized beam (at an incident angle of 84°). The spectra were collected with a resolution of 8 cm^−1^, averaged from 256 scans and baseline-corrected using OMNIC software (version 9). The molar masses (M_n_ and M_w_) of polymers were measured by gel permeation chromatography (GPC) using an instrument composed of an Agilent 1100 isocratic pump (Santa Clara, CA, USA), a degasser, a thermostatic box for columns, an auto-sampler, a MALS Dawn Heleos photometer (Wyatt Technology Corporation, Santa Barbara, CA, USA), and an Optilab T-rEX differential refractometer (Santa Barbara, CA, USA). The ASTRA 4.90.07 software (Wyatt Technology Corporation) was applied to data collection and processing. Two PLgel 5 μm MIXD-C columns were used for separation. The samples were diluted in DCM, while the volume of the injection loop was set to 100 μL. DCM was used as a mobile phase at a flow rate of 0.8 mL/min. The Dawn Heleos was calibrated using a polystyrene standard (M_n_ = 30,000 g/mol) in p.a. grade DCM. ^1^H NMR spectra were recorded by means of Bruker Avance III 600 MHz spectrometer in CDCl_3_.

### 2.3. Procedures

#### 2.3.1. Grafting of APTES-BPA Initiator

APTES-BPA initiator was synthesized on ITO and silicon surfaces. Silicon and ITO plates were sonicated in ethanol and then cleaned and activated by acidic or basic piranha solution. Silicon plates were immersed in acidic piranha (concentrated H_2_SO_4_ and 30% hydrogen peroxide mixed in a 1:3 volumetric ratio) for 15 min, while ITO plates in a basic piranha solution (30% ammonia and 30% hydrogen peroxide mixed in 1:1 volumetric ratio) at 50 °C. Afterwards, the samples were rinsed with copious amount of water, THF, and toluene and dried in the stream of argon. Finally, the plates were treated by hand-held air plasma cleaner (Reylon Plasma, Piezobrush® PZ2, Regensburg, Germany) for 1 min. Silicon or ITO plate was placed in 100 mL conical flask filled with 10 mL toluene and sealed with rubber septa. Next, the flask was purged with argon, and one droplet of APTES was added via syringe. The reaction proceeded overnight under argon atmosphere at room temperature. After completion, the plates were rinsed with an excess of toluene, sonicated for 10 min in toluene and finally rinsed with copious amount of DCM. The second step was carried out in an argon-purged system consisting of glass vessel and 100 mL conical flask stopped with rubber septa and connected with each other by double-tipped needle. Then, 20 μL of BPA-Cl in 2 mL of DCM were placed in the first vessel. APTES-modified plates were immersed in the mixture of 0.2 mL of TEA, and 10 mL of DCM in the second vessel. After passing argon through the reaction system for 15 min, the BPA-Cl mixture was transferred to the second vessel in the stream of the argon and the solution was left overnight to react. After completion, the initiator-modified plates were rinsed with DCM, sonicated in DCM (5 min), rinsed with an excess of methanol, and finally dried under a stream of argon. 

#### 2.3.2. Photoinduced Iron-Catalyzed Surface-Initiated ATRP of MMA

Surface-initiated photoinduced iron-catalyzed ATRP (SI-photo-Fe-ATRP) of MMA was performed with and without the addition of the sacrificial initiator—EBPA. The molar ratios of the reagents in case of the reactions performed with sacrificial initiator were as follows: [MMA]/[EBPA]/[FeBr_3_]/[TBABr] = 100/0.25/0.02/0.02. The polymerization mixture was prepared in the dark, in an argon-purged system consisting of amber glass vessel connected with double-tipped needle with specially designed polymerization vessel sealed with a rubber septum. Further, 4.5 mL of MMA (42.12 mmol) and 1.5 mL of anisole were added to amber glass vessel, while initiator-modified silicon plate and 18.5 μL of EBPA (0.1 mmol) were placed in the polymerization vessel. The system was purged for 10 min with argon and then 3 mL of degassed solution of FeBr_3_ (0.83 mg/mL; 0.008 mmol) and TBABr (0.9 mg/mL; 0.008 mmol) in anisole was added to the amber vessel via syringe. The whole reaction system was purged with argon for 5 min and then the mixture of MMA, anisole, FeBr_3_, and TBABr was transferred to the polymerization vessel in the stream of argon via double-tipped needle. Afterwards, the vessel was exposed to 450 nm LED light (Thorlabs) for a given time (see Figure S1). After polymerization, the silicon plate was rinsed with copious amount of toluene, sonicated in toluene, THF, and 0.1 M HCl to remove any impurities of the catalyst. Finally, it was rinsed with water and methanol and dried under a stream of argon. The procedure of SI-photo-Fe-ATRP of MMA in the absence of the sacrificial initiator (EBPA) was the same as well as molar ratios of the used reagents ([MMA]/[FeBr_3_]/[TBABr] = 100/0.02/0.02).

#### 2.3.3. Photoinduced Iron-Catalyzed ATRP of MMA in Solution

Photo-Fe-ATRP of MMA in solution was carried out for two different molar ratios of monomer to initiator: (1) [MMA]/[EBPA]/[FeBr_3_]/[TBABr] = 100/0.25/0.02/0.02 and (2) [MMA]/[EBPA]/[FeBr_3_]/[TBABr] = 100/1/0.02/0.02. The applied experimental procedure was the same as in case of SI-photo-Fe-ATRP but without the presence of initiator-modified plate in the polymerization vessel. Moreover, polymerization mixture was stirred during photo-Fe-ATRP in opposite to SI-photo-Fe-ATRP. Small portions of the reaction mixture were collected successively to track the MMA conversion using ^1^H NMR. The M_n_ and M_w_/M_n_ were determined by GPC measurements. Before GPC analysis, the collected samples were precipitated in the cold methanol, centrifuged, washed several times with cold methanol, once again centrifuged, and finally dried in vacuum oven at 40 °C.

#### 2.3.4. Photoreduction Kinetics of FeBr_3_ Catalyst

The polymerization solution of FeBr_3_, TBABr, and MMA in anisole (without the presence of the initiator) was irradiated by the LED lamp keeping light intensity and molar ratios of reagents the same as in case of polymerizations (see Section 2.3.2 and Section 2.3.3). After a certain time of irradiation, small aliquots were collected in the dark, diluted (30 times) by anisole under argon atmosphere, and immediately measured by UV–VIS spectroscopy in transmittance mode.

## 3. Results and Discussion 

### 3.1. Grafting of APTES-BPA Initiator

**Scheme 2:**
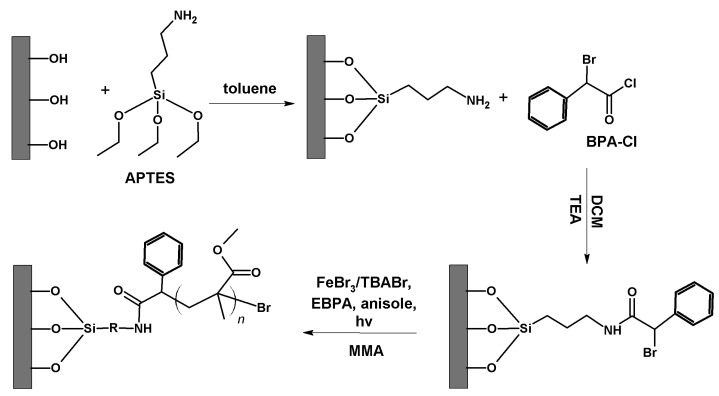
Synthesis of poly(methyl methacrylate) (PMMA) brushes via surface-initiated photoinduced iron-catalyzed-ATRP (SI-photo-Fe-ATRP).

Monolayer of APTES-BPA on ITO or silicon oxide surface was synthesized via two-step procedure, as schematically depicted in Scheme 2. 

In the first step, APTES was grafted on the precleaned surfaces. The grafting of APTES on IR-reflective ITO was verified by means of grazing-angle FTIR spectroscopy (see Figure S2). The appearance of characteristic bands of N-H deformative vibrations (1592 cm^−1^) and C-H stretching vibrations (2974 and 2947 cm^−1^) confirmed successful modification of ITO with APTES molecules [5]. In the second step, APTES-modified substrate was subjected to reaction with α-Bromophenylacetyl chloride (BPA-Cl). The covalent attachment of BPA to APTES and hence successful formation of APTES-BPA initiator was confirmed by the IR bands observed at 1642 cm^−1^ (amide I, C=O stretching vibrations) and 1542 cm^−1^ (amide II, N-H bending vibrations). Furthermore, the appearance of the band at 3020 cm^−1^ (C-H stretching in aromatic ring) additionally supports the realization of the reaction. Although, the initiator grafting was confirmed on IR-reflective ITO, the surface chemistry is the same as in the case of silicon wafers (transparent to IR light) [38] that are not compatible with the used grazing-angle reflectance FTIR accessory.

### 3.2. Surface-Initiated Photoinduced Iron-Catalyzed ATRP of MMA

**Scheme 3:**
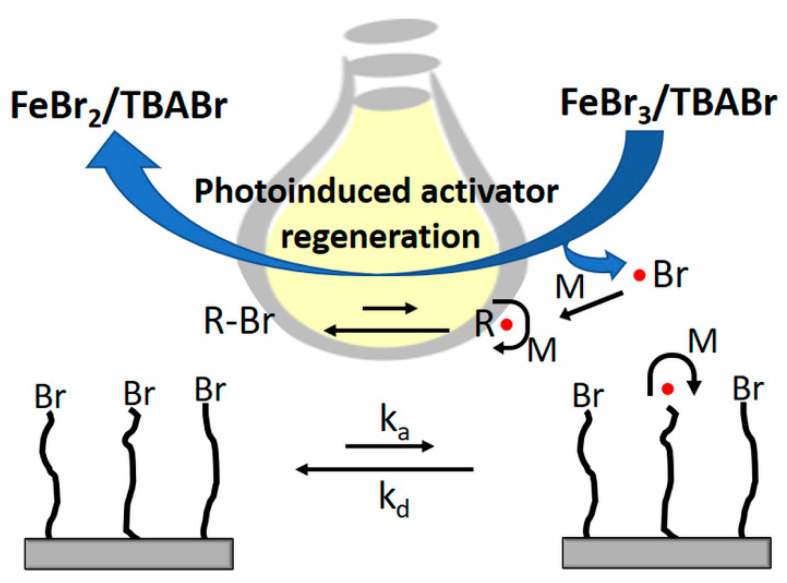
Surface-initiated photoinduced iron-catalyzed ATRP.

Photo-Fe-ATRP is commonly conducted under high energetic UV light using high concentrations of iron(III)-based catalyst [17,33,34]. In 2017, Dadashi-Silab et al. have shown that addition of TBABr ligand enables reduction in FeBr_3_ concentration to only 100–400 ppm against monomer and synthesis of PMMA with low dispersity under visible light [37]. It is worth emphasizing that both FeBr_2_ and FeBr_3_ may occur in the active ionic forms during ATRP or atom transfer radical addition (ATRA) [39], depending on the nature of the solvent and/or ligand [40]. Schroeder et al. demonstrated that (Fe^II^Br_3_(solvent))^−^/(Fe^III^Br_4_)^−^ species actually mediate the ATRP equilibrium when the iron catalyst is used in equimolar ratio to TBABr [41]. Furthermore, it was found that stabilization of the activator (Fe^II^Br_3_(solvent))^−^ is enhanced in nonpolar solvents such as anisole leading to the higher activation rate constants (k_act_) with respect to the polar solvents. Dadashi-Silab et al. reported that TBABr additionally stabilizes Fe(III) and Fe(II) species in anisole (nonpolar solvent) in case of photo-Fe-ATRP improving the control over the polymerization [37]. According to that, we have developed the conditions for controlled growth of PMMA brushes by means of SI-photo-Fe-ATRP with ppm levels of the catalyst. For simplicity, we abbreviated the activator as FeBr_2_/TBABr, while deactivator as FeBr_3_/TBABr. The polymerizations were conducted under visible light irradiation (λ= 450 nm). In order to initiate the photo-ATRP, FeBr_3_/TBABr has to be photoreduced to its active form—FeBr_2_/TBABr. An activator may then react with surface-grafted initiators leading to the formation of active radical centers propagating with monomers as well as regeneration of FeBr_3_/TBABr (see Scheme 3).

Therefore, at first, we determined the time necessary to reduce the FeBr_3_/TBABr catalyst to its active form using time-resolved UV–VIS measurements (Figure S3a). Typical polymerization mixture without both sacrificial initiator and initiator-modified surface was irradiated with 450 nm LED light under argon and then UV–VIS spectra after certain time intervals were recorded. As previously observed by Dadashi-Silab et al. [37], FeBr_3_ in the presence of MMA and TBABr is quickly reduced (after 15–20 min), which is confirmed by the disappearance of the absorption band with maximum at 475 nm. Very slow reduction ofthe catalyst was observed when the polymerization mixture was exposed to ambient light, while no reduction was noticed in the dark (see Figure S3b) 

**Figure 1:**
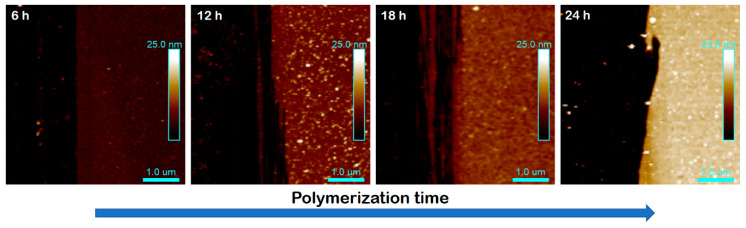
AFM topography images at the edge of the scratches performed in poly(methyl methacrylate) (PMMA) brushes obtained after different times of surface-initiated photoinduced iron-catalyzed atom transfer radical polymerization (SI-photo-Fe-ATRP) in the presence of the sacrificial initiator.

The brush growth was followed by performing a series of polymerizations in the presence of the sacrificial initiator (target degree of polymerization N = 400) keeping the same conditions but varying the polymerization time. The dry brush thicknesses (h_dry_) were measured by atomic force microscopy (AFM) in at least three different locations on each sample (see the experimental part and Figure 1). In order to calculate the grafting density for selected reactions (Table 1, entry 5 and 6), the molar masses of the polymers produced simultaneously in the solution above the grafting surface were analyzed. 

The reactions were conducted under irradiation with 450 nm LED light. Conditions were [MMA]/[EBPA]/[FeBr_3_]/[TBABr] = 100/0.25/0.02/0.02. Conversions were calculated by ^1^H NMR. Theoretical molecular weights (M_nth_) were calculated using the following equation: M_nth_ = M_EBPA_ + [MMA]/[EBPA] × conversion × M_MMA_. Dispersity (M_w_/M_n_) and number-average molecular weights (M_n_) were determined by GPC measurements. M_α_ was calculated from the degree of polymerization (N) of surface-grafted PMMA chains estimated based on Equation (3). 

The AFM topography images of the obtained samples (Figure 1) confirmed the formation of continuous and homogenous films. The bright spots on topography images indicate deposition of some physically adsorbed macromolecules and/or covalently attached chains from a solution via locally occurred recombination reactions. The brush grafting density (Σ) was calculated in two ways: (1) using M_n_ values of free polymers in solution (M_n_ = 20500, M_w_/M_n_ = 1.55 Table 1 entry 5) obtained by GPC measurements (Equation (1), Σ_GPC_) and (2) by determining the swelling ratio (α = h_wet_/h_dry_) of PMMA brushes in the good solvent—toluene (Equation (2), Σ_α_), according to the methodology described previously [38].

(1) $$\Sigma_{\mathrm{GPC}}=\frac{h_{\mathrm{dry}}\times \rho \times N_{A}}{M_{n}}$$

where ρ = 1.18 × 10^−21^ g/nm^3^—PMMA density and N_A_—Avogadro constant (chains/mol).

(2) $$\Sigma_{\alpha }={\left(\frac{1.08}{\alpha }\right)}^{\frac{3}{2}}$$

As the brush grafting density should not change with polymerization time, the calculations were performed only for selected samples. The calculated values are as follows: Σ_GPC_ = 0.62 chains/nm^2^ and Σ_α_ = 0.40 chains/nm^2^ (Table 1, entry 5). The Σ_GPC_ value was additionally calculated for the thickest brushes (Table 1, entry 6), showing comparable results Σ_GPC_ = 0.60 chains/nm^2^. Therefore, Σ_GPC_ was found to be 1.5 times higher than Σ_α_ pointing to some differences in polymerization kinetics of photo-Fe-ATRP and SI-photo-Fe-ATRP (see further). 

**Figure 2:**
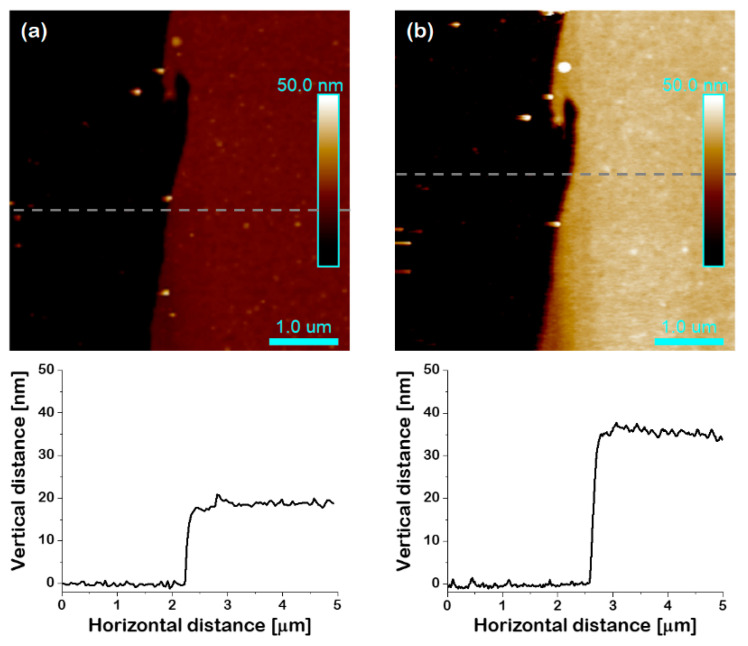
AFM topography images of PMMA brush (Table 1, entry 5) in (**a**) dry and (**b**) wet state (measurements performed in toluene).

**Figure 3:**
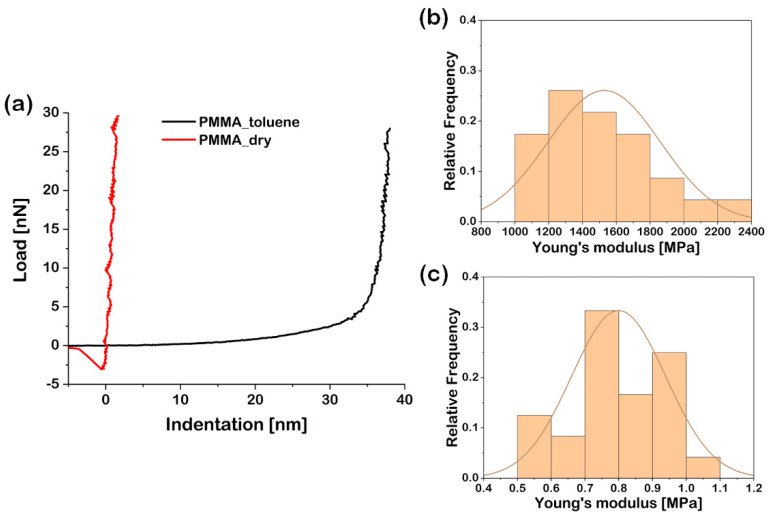
Exemplary AFM nanoindentation curves of (**a**) PMMA in the air and toluene with histograms showing distribution of calculated Young’s moduli for (**b**) PMMA brush in the air and (**c**) the same brush immersed in toluene.

The wet brush thickness for calculation of Σ_α_ was obtained by means of AFM measurements in toluene. The dry brush thicknesses as well as wet thickness in toluene were measured for the sample after 24 h of polymerization (Table 1, entry 5) in the same place (see Figure 2). Liquid measurements were performed with soft probes (spring constant equal to 0.97 ± 0.05 N/m) at low loads (around 200 pN) in order to the keep minimal sample deformation. The measured wet thickness 36 ± 2 nm was also confirmed by nanoindentation measurements (see Figure 3). 

The nanoindentation measurements were performed in the dry and wet state. A series of indentation curves were captured in nearly the same locations on the sample surface in different conditions using the same tip. In case of dry samples, the Hertz model was fitted to the approach curves in the range of 0–25 nN load, which corresponded to the indentation depths below 2 nm avoiding contribution of the underlying substrate. The same model was also applied to the brushes in the wet state; however, the model was fitted only in the range of 0–0.5 nN keeping penetration depths below 5 nm. Exemplary approach indentation curves are presented in Figure 3a. Applying the same load (30 nN) to the dry and wet samples resulted in measuring substantially different indentation depths for both samples. It proves that PMMA brushes in toluene are highly swollen. The obtained penetration depths for the dry PMMA brush was in the range of 1–2 nm, while in case of the brush in toluene 35–37 nm until reaching the hard silicon surface (vertical slope of the curve at loads above 25 nN). These results confirm the accuracy of the measurement of wet brush thickness by AFM topography scanning in toluene (Figure 2 and Table 1 entry 5). Figure 3b,c shows histograms of the calculated Young’s modulus for both samples. PMMA brushes in the wet state demonstrated around 3–4 orders of magnitude lower Young’s modulus (8 × 10^−4^ ± 1 × 10^−4^ GPa) compared to the brushes in the dry state (1.5 ± 0.3 GPa). The Young’s modulus in the dry state might be slightly underestimated as the values obtained before for bulk PMMA (2–3 GPa) [42] and thick PMMA brushes (~8 GPa) were even higher [43]. On the other side, Wang et al. reported smaller values of Young’s modulus (0.76 GPa) for dry PMMA brushes [44]. The observed discrepancies could be related to the application of quite soft cantilever for the measurements (see experimental) that are less accurate for hard samples. However, we aimed to show the comparison of brush thickness and stiffness in wet (very soft) and dry state (hard film) using one tip under the same conditions. 

### 3.3. Comparison of Kinetics of SI-Photo-Fe-ATRP and Photo-Fe-ATRP

**Figure 4:**
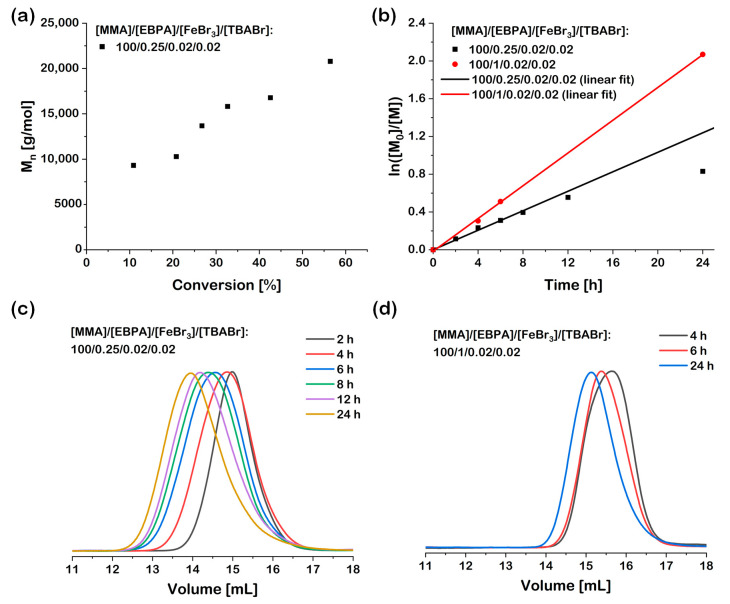
Results of syntheses of PMMA polymers in the solution by means of photo-Fe-ATRP: (**a**) number-average molecular weight (Mn) vs. conversion, (**b**) semilogarithmic kinetic plots, (**c**,**d**) gel permeation chromatography (GPC) chromatograms.

In order to compare the kinetics of surface-initiated process with polymerization in the solution, photo-Fe-ATRP was conducted in the same conditions as mentioned above. The evolution of molar masses and monomer conversion were followed by means of GPC and ^1^H NMR measurements (see Table 2 and Figure 4). The obtained semilogarithmic kinetic plot showed near linear characteristics with some deviations for longer reaction time. 

The reactions were conducted under 450 nm LED light. Monomer conversions were calculated using ^1^H NMR. Theoretical molecular weights (M_nth_) were calculated using following formula: M_nth_ = M_EBPA_ + [MMA]/[EBPA] × conversion × M_MMA_. Dispersity (M_w_/M_n_) and number-average molecular weights (M_n_) were determined by GPC measurements. 

Besides quite linear kinetics characteristics, the molecular weight distributions of the obtained polymers at longer reaction times were rather broad. It is presumably associated with initiation of polymerization not only from the initiator molecules but also by the 2,3-dibromoisobutyrate species produced during the reaction of the catalyst with monomer as it was reported by Matyjaszewski et al. [17,37] (see also Scheme 3). It is supported also by the obtained GPC chromatograms (see Figure 4c) that present some broadening of the peak with the polymerization time and hence formation of short chains with lower molar masses. One cannot also exclude terminations at longer reaction times, as indicated by decreasing concentration of the growing macroradicals, manifested by deviations in the kinetic plot (see Figure 4b). Rolland et al. have shown that dispersity in case of photo-Fe-ATRP could be modified by changing the catalyst concentration as well as ATRP initiator content (target N) [45]. It was found that dispersions of the formed polymers were increasing with decreasing FeBr_3_ and TBABr concentration. Interestingly, much lower dispersions were reported for polymerizations with target N = 20 (M_w_/M_n_ = 1.10) compared to N = 150 (M_w_/M_n_ = 1.50) when FeBr_3_ concentrations were very low (below 50 ppm). Therefore, in order to reduce the probability of undesirable initiation of ATRP from monomer molecules, we have performed photo-Fe-ATRP with a smaller target degree of polymerization (N = 100), while keeping other conditions constant. As expected, the apparent propagation constant and monomer conversions increased substantially (Figure 4b), while the obtained dispersions were much lower demonstrating better control over the growing polymers (Table 2 and Figure 4d).

**Figure 5:**
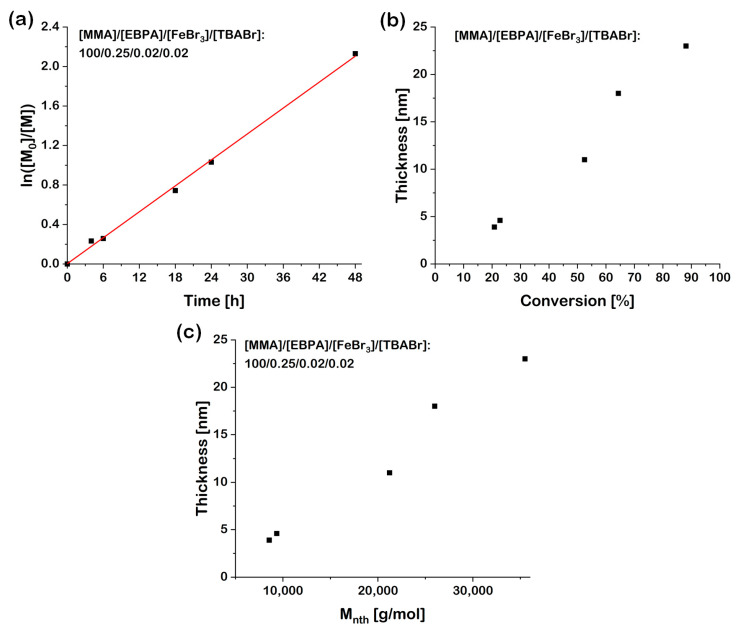
Results of syntheses of PMMA brushes by means of SI-photo-Fe-ATRP with sacrificial initiator: (**a**) semilogarithmic kinetic plot for synthesis of PMMA brushes (based on ^1^H NMR analysis of polymerization solution after SI-photo-Fe-ATRP), (**b**) thickness vs. monomer conversion (calculated based on ^1^H NMR analysis of polymerization solution after SI-photo-Fe-ATRP), and (**c**) thickness vs. theoretical number-average molecular weight.

In case of the brushes synthesized in the presence of sacrificial initiator, the monomer conversion was analyzed by ^1^H NMR spectroscopy to compare the kinetics of surface-initiated process with photo-Fe-ATRP. The dependences of dry brush thickness (proportional to molecular masses of grafted polymers) against monomer conversion and M_nth_ (calculated from ^1^H NMR) show linear characteristic (Figure 5b,c). Moreover, the linear semilogarithmic kinetic plot was obtained indicating the controlled characteristics of the polymerization (Figure 5a). Interestingly, the dispersity of free PMMA in case of SI-photo-Fe-ATRP with sacrificial initiator was much lower (1.54–1.55, Table 1 entry 5 and 6) compared to photo-Fe-ATRP, pointing on better control of polymerization. The only differences between those two polymerizations were associated with the presence of initiator-modified plate and no stirring of the polymerization solution in case of SI-photo-Fe-ATRP. It is worth to emphasize that M_n_ values after the same reaction time (24 h) were almost identical in both experiments (see Table 1 entry 5 and Table 2 entry 6). Recently, we have shown that the degree of polymerization (N) in case of PMMA brushes could be estimated using Equation (3) [38].

(3) $$h_{\mathrm{dry}}=0.141{N\Sigma }_{\alpha }$$

Calculating N, one may easily estimate the molar mass (M_α_) of surface-grafted chains just by multiplying N by the molar mass of monomer. Assuming the same grafting density of the brushes obtained after various times of polymerization, molar masses of the grafted chains were calculated as presented in Table 1.

**Figure 6:**
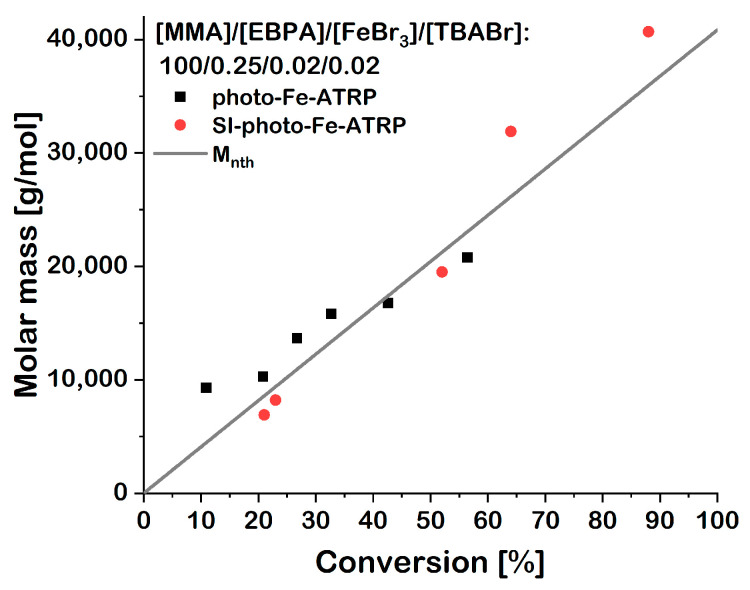
Plot presenting dependency of estimated M_α_ of grafted PMMA brushes and M_n_ of PMMA polymers obtained in solution during photo-Fe-ATRP vs. monomer conversion (conversions calculated by means of ^1^H NMR).

Figure 6 shows the dependence of calculated molar masses (M_α_) of surface-grafted chains (based on Table 1) and M_n_ values of polymers obtained by photo-Fe-ATRP (based on Table 2) against monomer conversion. This graph shows that at lower conversions and hence shorter reaction times, the molecular masses of grafted chains are lower than in case of polymers in the solution. Interestingly, the increase in molar masses at longer reaction times for PMMA brushes is greater compared to the polymers formed in the solution. We observed similar dependency in “classical” surface-initiated ATRP of MMA [38]. Moreover, the observed differences seem to explain the overestimated grafting density calculated based on GPC measurements. It is worth emphasizing that evolution of molar masses with the monomer conversion in case of SI-photo-Fe-ATRP is consistent with the linear dependence of M_nth_ on the conversion, while for photo-Fe-ATRP, larger deviations were observed, already for small conversions. The observed differences between SI-photo-Fe-ATRP and photo-Fe-ATRP could be also associated with a slightly different structure of APTES-BPA and EBPA initiators that may lead to some distinction in activation rate constants.

### 3.4. The Influence of Sacrificial Initiator on SI-Photo-Fe-ATRP Kinetics

In order to maintain the controlled characteristics of “classical” surface-initiated ATRP, it is required to keep the appropriate ratio of deactivator to activator, shifting the K_ATRP_ towards deactivation. It could be achieved by the addition of deactivator or sacrificial initiator. In case of SI-photo-Fe-ATRP, the deactivator (FeBr_3_/TBABr) is reduced during the first 15–20 min, while it can be regenerated by halogen transfer from the initiator or dormant chain to activator. However, the concentration of the initiator grafted on the flat silicon wafer is not sufficient to effectively regenerate the deactivator. Therefore, SI-photo-Fe-ATRP without sacrificial initiator may not demonstrate controlled characteristics. Surprisingly, the linear brush growth was observed during the first 6 h of the reaction. The deviation from the linear growth was observed only for long reaction time (24 h). Furthermore, increased viscosity of polymer solution and quite significant variations in layer thickness (after 24 h of polymerization) were detected in different places on the sample indicating not negligible monomer conversion and less controlled process. The Matyjaszewski group reported that photo-Fe-ATRP could be initiated even without the addition of halogen initiators [17,37]. During photoreduction of FeBr_3_, photolysis of Fe-Br bond enables regeneration of the activator and production of Br· radicals (see Scheme 3) [40]. As a result, halogen initiator could be formed in situ via photoreduction of FeBr_3_/TBABr in the presence of methyl methacrylate leading to 2,3-dibromoisobutyrate that can further initiate the polymerization. Interestingly, the polymerizations conducted without “classical” ATRP initiator (EBPA) can proceed, but with a long induction period (few hours) necessary to form initiating sites as described above [17,37]. It explains the linear brush growth at shorter times in case of surface-initiated polymerization without sacrificial initiator, due to negligible monomer conversion and hence almost constant concentration of the monomer. However, the concentration of 2,3-dibromoisobutyrate species right after photoreduction seems to be sufficient to partially regenerate the deactivator achieving controlled characteristics of the surface-initiated process. Moreover, the observed delayed initiation from 2,3-dibromoisobutyrate [17,37], which additionally should demonstrate lower activation rate constant compared to EBPA [46], clarifies an increase in M_w_/M_n_ values with the reaction time of photo-Fe-ATRP. It is reflected in broadening of GPC peaks from the side of longer retention times and hence formation of fractions of polymers with lower molar masses. Therefore, in order to obtain polymer with narrow molar mass distribution one has to increase the concentration of classical halogen initiator (low N values) or just resign from using it at the expense of lower monomer conversions as described elsewhere [17]. 

**Figure 7:**
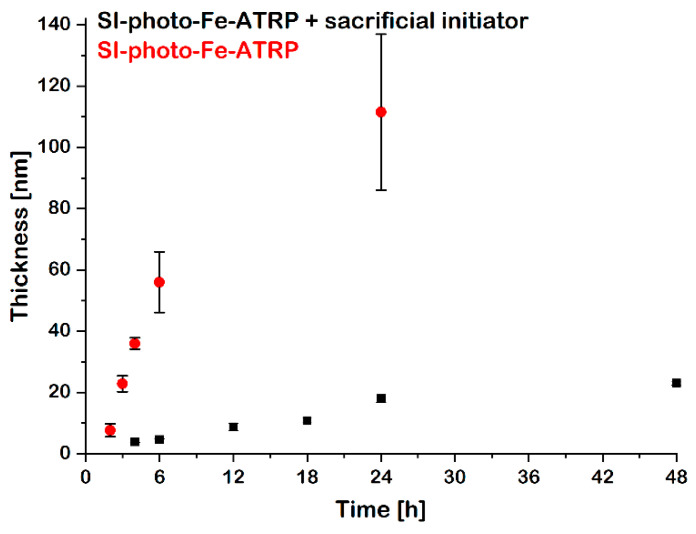
Brush thickness evolution with time of SI-photo-Fe-ATRP in the presence (black color) and without (red color) sacrificial initiator.

The comparison of the kinetics of SI-photo-Fe-ATRP with and without sacrificial initiator revealed substantial differences in the polymerization rate (Figure 7). Around 10 times thicker layers were obtained during the first 6 h of reaction in case of polymerization without sacrificial initiator compared to the reaction with EBPA (Figure 7). The in situ generation of ATRP initiator (2,3-dibromoisobutyrate) during photoreduction of FeBr_3_/TBABr in the presence of MMA helps to maintain the control by initiation of polymerization in the solution and hence regeneration of the deactivator. As a result, the simplified reaction system could be used with ppm amount of the catalyst without free initiator in the solution. It is worth emphasizing that the developed conditions enable the formation of very thick layers of PMMA brushes at room temperature, which is of great importance as such materials are commonly synthesized at elevated temperatures [38,47,48]. 

## 4. Conclusions

Surface-initiated photoinduced iron-catalyzed ATRP was developed and used for the first time for controlled synthesis of PMMA brushes. Semilogarithmic kinetics plots as well as the evolution of molar masses of grafted macromolecules with monomer conversion indicate on controlled characteristics of SI-photo-Fe-ATRP. It is worth to mention that all the studied polymerizations were conducted under visible light (illuminated with 450 nm LED light) with only 200 ppm of FeBr_3_/TBABr catalyst. The grafting density of PMMA brushes was determined in two ways confirming the formation of dense layers. The comparison of the kinetics of SI-photo-Fe-ATRP and photo-Fe-ATRP (conducted under the same conditions) revealed obtaining of smaller molar masses of grafted chains at low monomer conversions, while higher for high conversions compared to polymers generated in the solution. The addition of sacrificial initiator to SI-photo-Fe-ATRP substantially slows down the brush growth with respect to the reactions performed without additional initiators. Interestingly, simplified SI-photo-Fe-ATRP (without sacrificial initiator) preserve controlled characteristics up to 6 h (low monomer conversion) of the process owing to in situ formation of halogen initiator by photoreduction of FeBr_3_/TBABr enhanced by methyl methacrylate that can further initiate the polymerization in solution and regenerate the deactivator (FeBr_3_/TBABr). Long reaction times lead to higher monomer conversion, an increase in polymerization mixture viscosity, and hence the slow growth of the brushes. It is worth underlying that simplified SI-photo-Fe-ATRP enables formation of thick PMMA films at room temperature with ppm levels of biocompatible catalyst. The developed technique could be potentially extended over other methacrylate monomers as it was demonstrated in the case of photo-Fe-ATRP in solution [37]. SI-photo-Fe-ATRP could be recognized as a facile synthetic methodology for formation of functional polymeric coatings for electronic and bioapplications that require restrictive reaction conditions free of toxic transition metal compounds.

## Figures and Tables

**Table 1:** Results of syntheses of poly(methyl methacrylate) (PMMA) brushes via surface-initiated photoinduced iron-catalyzed atom transfer radical polymerization (SI-photo-Fe-ATRP) with the sacrificial initiator.

Entry	Time (h)	h_dry_ (nm)	M_n_	M_w_/M_n_	Conversion (%)	M_nth_	M_α_
1	4	3.9 ± 0.3	–	–	21	8600	6900
2	6	4.6 ± 0.4	–	–	23	9400	8200
3	12	8.7 ± 1.2	–	–	–	–	–
4	18	11.0 ± 0.7	–	–	52	21,200	19,500
5	24	18.0 ± 1.9	20,500	1.55	64	26,000	31,900
6	48	23.0 ± 1.6	27,500	1.54	88	35,500	40,700

**Table 2:** Results of syntheses of PMMA polymers in the solution by photo-Fe-ATRP.

Entry	Time (h)	M_n_ (g/mol)	M_nth_ (g/mol)	Conversion (%)	M_w_/M_n_
[MMA]/[EBPA]/[FeBr_3_]/[TBABr] = 100/0.25/0.02/0.02					
1	2	9300	4600	11	1.33
2	4	10,300	8600	21	1.63
3	6	13,700	10,900	27	1.67
4	8	15,800	13,300	33	1.78
5	12	16,800	17,300	43	1.90
6	24	20,800	22,800	56	1.96
[MMA]/[EBPA]/[FeBr_3_]/[TBABr] = 100/1/0.02/0.02					
7	4	5200	2900	26	1.30
8	6	5800	4200	40	1.29
9	24	7000	9000	87	1.41

## Supplementary Materials

The following are available online at https://www.mdpi.com/1996-1944/13/22/5139/s1, Figure S1: Photograph of the photopolymerization system; Figure S2: FTIR spectra of (3-aminopropyl) trimethoxysilane (APTES) and APTES-α-bromophenylacetyl chloride (BPA)-modified ITO plate; Figure S3: UV–VIS spectra of the polymerization mixture ([MMA]/[FeBr_3_]/(tetrabutylammonium bromide catalyst [TBABr]) = 100/0.02/0.02): (a) after various irradiation times with 450 nm LED light and (b) exposed to ambient light and kept in darkness. The polymerization mixture was diluted 30 times before the measurements ([FeBr_3_] = 0.03 mM).
