# Supplementary material for: Surface-Initiated Photoinduced Iron-Catalyzed Atom Transfer Radical Polymerization with ppm Concentration of FeBr3 under Visible Light
Source: Materials (Basel). 2020 Nov 14;13(22):5139. doi: 10.3390/ma13225139 (PMC7697009; doi:10.3390/ma13225139)
Supplement: Supplementary file 1 [file materials-13-05139-s001.pdf]

materials

Supplementary Materials

Surface-Initiated Photoinduced Iron-Catalyzed Atom Transfer Radical Polymerization with ppm Concentration of $FeBr_3$ under Visible Light

Monika Słowikowska [1], Kamila Chajec [1], Adam Michalski [1,2], Szczepan Zapotoczny [1] and Karol Wolski [1,*]

1 Faculty of Chemistry, Jagiellonian University, Gronostajowa 2, 30-387 Kraków, Poland; slowikowska@chemia.uj.edu.pl (M.S.); kamila.chajec@spoko.pl (K.C.); michadam@cbmm.lodz.pl (A.M.); zapotocz@chemia.uj.edu.pl (S.Z.)
2 Center of Molecular and Macromolecular Studies, Polish Academy of Sciences, Sienkiewicza 112, 90-363 Lodz, Poland
* Correspondence: wolski@chemia.uj.edu.pl

Received: 27 October 2020; Accepted: 12 November 2020; Published: 14 November 2020

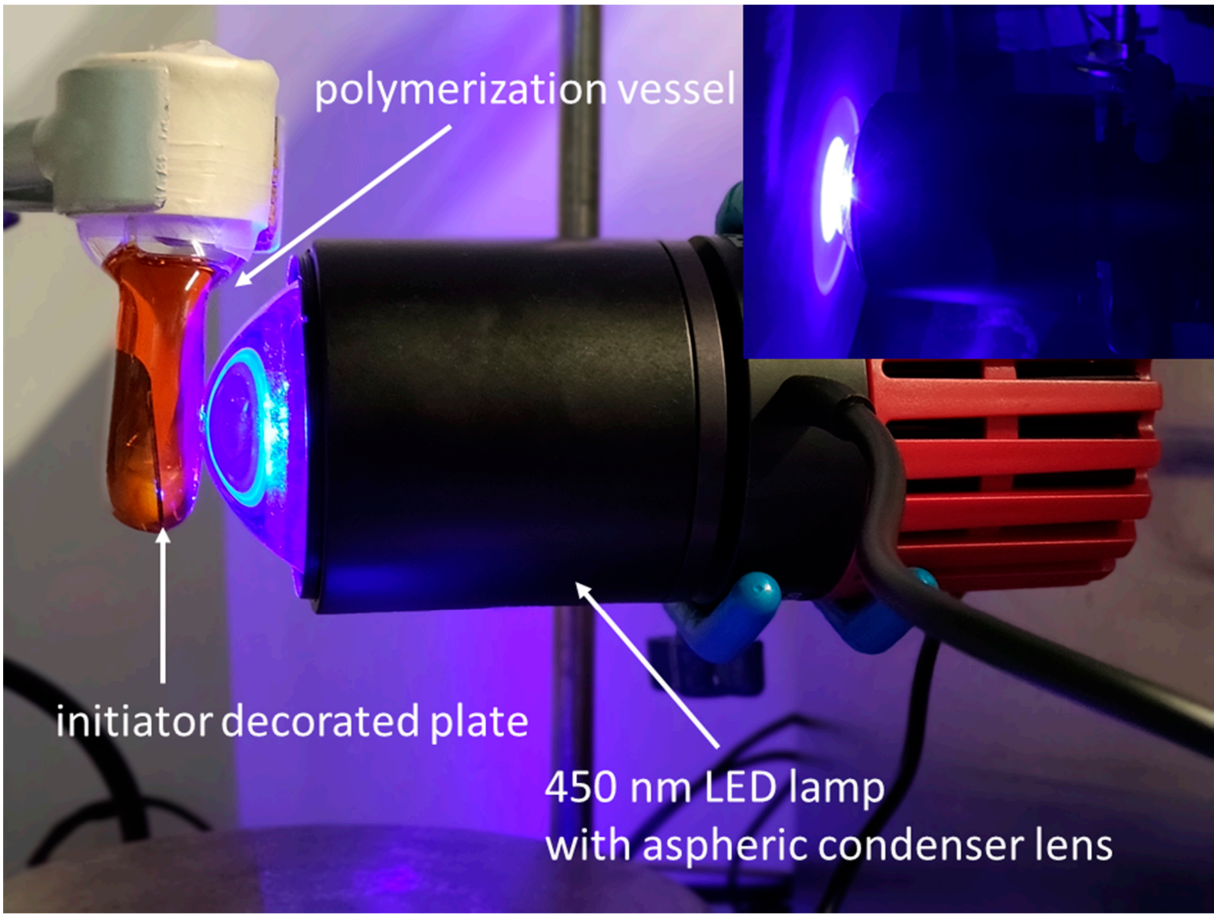

Figure S1. Photo of the reaction system.

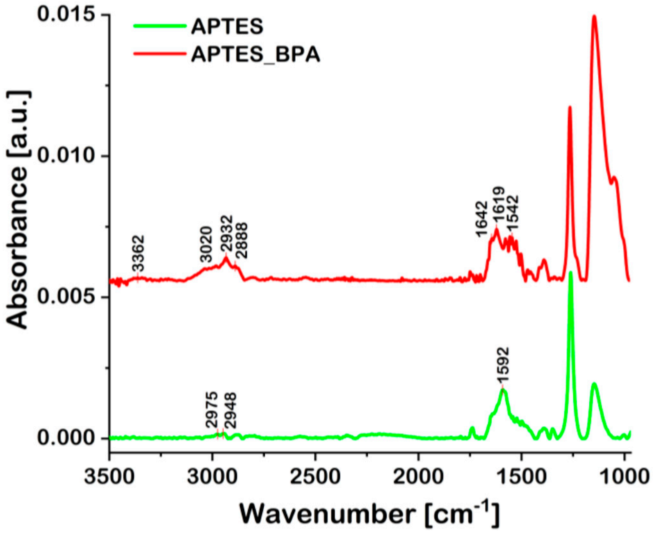

Figure S2. FTIR spectra of APTES and APTES-BPA modified ITO plate.

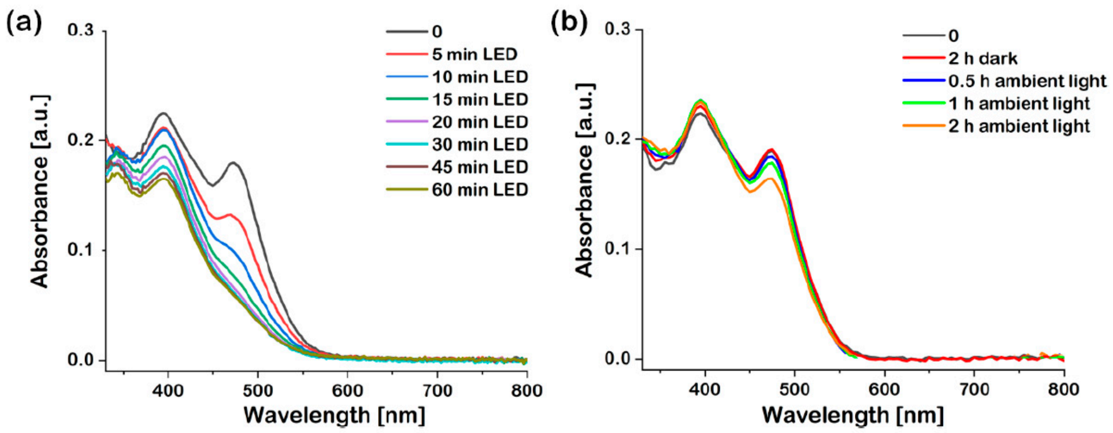

Figure S3. UV-VIS spectra of the polymerization mixture ([MMA]/[$FeBr_3$]/[TBABr] = 100/0.02/0.02): (**a**) after various irradiation times with 450 nm LED light, and (**b**) exposed to ambient light and kept in darkness. The polymerization mixture was diluted 30 times before the measurements ([$FeBr_3$] = 0.03 mM).
